# Biochemical and biomechanical characterization of an autologous protein-based fibrin sealant for regenerative medicine

**DOI:** 10.1007/s10856-024-06780-4

**Published:** 2024-03-08

**Authors:** Eduardo Anitua, Ander Pino, Roberto Prado, Francisco Muruzabal, Mohammad Hamdan Alkhraisat

**Affiliations:** 1 University Institute for Regenerative Medicine and Oral Implantology (UIRMI), Vitoria, Spain; 2 BTI-Biotechnology Institute, Vitoria, Spain

## Abstract

Accidental events or surgical procedures usually lead to tissue injury. Fibrin sealants have proven to optimize the healing process but have some drawbacks due to their allogeneic nature. Autologous fibrin sealants present several advantages. The aim of this study is to evaluate the performance of a new autologous fibrin sealant based on Endoret®PRGF® technology (E-sealant). One of the most widely used commercial fibrin sealants (Tisseel®) was included as comparative Control. E-sealant´s hematological and biological properties were characterized. The coagulation kinetics and the microstructure were compared. Their rheological profile and biomechanical behavior were also recorded. Finally, the swelling/shrinkage capacity and the enzymatic degradation of adhesives were determined. E-sealant presented a moderate platelet concentration and physiological levels of fibrinogen and thrombin. It clotted 30 s after activation. The microstructure of E-sealant showed a homogeneous fibrillar scaffold with numerous and scattered platelet aggregates. In contrast, Control presented absence of blood cells and amorphous protein deposits. Although in different order of magnitude, both adhesives had similar rheological profiles and viscoelasticity. Control showed a higher hardness but both adhesives presented a pseudoplastic hydrogel nature with a shear thinning behavior. Regarding their adhesiveness, E-sealant presented a higher tensile strength before cohesive failure but their elastic stretching capacity and maximum elongation was similar. While E-sealant presented a significant shrinkage process, Control showed a slight swelling over time. In addition, E-sealant presented a high enzymatic resorption rate, while Control showed to withstand the biodegradation process in a significant way. E-sealant presents optimal biochemical and biomechanical properties suitable for its use as a fibrin sealant with regenerative purposes.

**Graphical Abstract:**

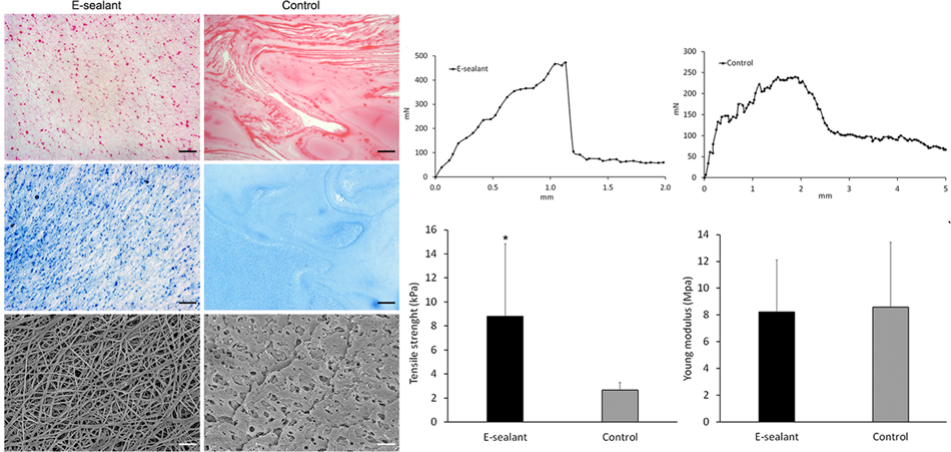

## Introduction

Accidental events or surgical procedures usually lead to tissue injury that require medical intervention aimed at accelerating hemostasis and restoring the native biological integrity and function. Clips, sutures, staples or strips are the most extended medical devices to promote wound closure in these situations [1]. However, injuries that have not undergone precise closing or experience excessive tensile strength can lead to scarring and pain. Sometimes, these methods are not sufficient to prevent the leaking of biological fluids and can be challenging and time consuming especially over not readily accessible regions. Moreover, the relatively high infection rate and inconvenience in handling are recurrent drawbacks of these conventional procedures [2].

In the last decades tissue adhesives have gained the attention of clinicians as an alternative approach for wound management. This type of sealants can be rapidly applied in a minimally invasive manner with no need of deep expertise and are less prone to cause infections or damage delicate tissue [3]. Nowadays, there are different commercially available tissue adhesives based either on natural or synthetic polymers [4]. In this sense, fibrin-based tissue adhesives are sealants of natural polymer origin that are widely used in various medical fields including cardiovascular, orthopedic, ophthalmologic, and dermatologic surgery [5].

Mainly derived from pooled human plasma, fibrin sealants consist of fibrinogen and thrombin, two key components involved in the coagulation cascade [6]. These formulations are biocompatible and have proven to optimize the natural clotting, effectively stopping bleeding, and creating a barrier that helps in the healing process. This reduces the overall operating time of a procedure and minimizes the need for additional sutures [7]. However, allogeneic fibrin adhesives are not exempt from downsides, and they must be remembered when considering their clinical use. They can be relatively expensive compared to other methods due to the production process, which involves complex purification steps and stringent quality control. In addition, although efforts are made to ensure their safety through careful screening and testing, there is still a minimal risk of infectious disease transmission within isolated batches [8]. Moreover, fibrin sealants have a limited shelf life, and their effectiveness decreases over time. Therefore, they must be stored properly and used within their designated expiration date to ensure optimal performance. This factor requires careful management and monitoring of inventory to avoid wastage. In addition, while fibrin sealants are generally well-tolerated, they include excipients such as antifibrinolytic agents that may induce allergic reactions in some cases [9].

As an alternative to allogeneic and commercially available fibrin sealants, scientists are trying to develop new protocols based on patient´s own blood with the aim of developing autologous fibrin glues [6, 10, 11]. Many of these approaches begin with the obtention of the platelet-rich plasma fraction (PRP) after blood centrifugation. These sealants present some advantages compared to allogeneic ones including their biocompatibility and safety, as they significantly reduce the risk of disease transmission and allergic reactions. In addition, given their autologous nature they present reduced immunogenicity, minimizing the potential of immune-mediated complications [12]. In contrast to allogeneic glues, autologous fibrin sealants have been found to promote faster and more efficient wound healing. This is related to the presence not only of fibrinogen and thrombin components, but also a myriad of growth factors and cytokines released by platelets. This increases the chances of tissue regeneration because, apart from a fibrin scaffold, cells within the target tissue will receive biological signals that will trigger specific molecular pathways aimed at promoting wound healing [10].

However, there is a lack of standardization when facing PRP or autologous fibrin glue obtention methods. Nowadays, different preparation protocols and systems are available (speed/number of spin cycles, protein concentration yield and methodology, etc). This results in significant differences in their characteristics as determined by the platelet/protein concentration, leukocyte inclusion, presence and type of anticoagulant or platelet activation method [13]. In this sense, plasma rich in growth factors technology (Endoret®PRGF®) is a specific type of standardized PRP that is leukocyte-free and moderately enriched in platelets [14]. It is classified as pure-PRP [15], specifically P2-x-Bβ category and 24-00-11 according to two extensively proposed classifications [16–19]. Endoret®PRGF® has been widely applied in different medical fields including traumatology, ophthalmology, maxillofacial surgery and dermatology [16–19], and the main preparation protocol has not suffered from major changes over the years [20]. Moreover, the versatility of this technology allows the easy and fast in situ obtention of custom-tailored pharmaceutical formulations for different biomedical applications such as eye drops, a 3D-gel, a transparent membrane, or a topical ointment [21–24].

In this line, a new fibrin sealant based on Endoret®PRGF® has been recently developed with the aim of overcoming the inherent limitations of autologous fibrin glues such as slower clotting time or reduced adhesive strength. This novel formulation will subsequently be referred to as “E-sealant”. In the present study, the biochemical and biomechanical performance of E-sealant has been compared against one of the most extended and widely used fibrin sealants in the market.

## Materials and methods

### E-sealant preparation

As E-sealant is an autologous formulation with no additives or excipients, it must be prepared just prior application with the aim of maintaining its adherence and biomechanical properties. Thus, for each in vitro assay, freshly prepared E-sealant was obtained following the Endoret®PRGF® protocol described previously [14] with variations, as follows.

After informed consent obtention, blood from three to five healthy volunteers was harvested into 9 mL collection tubes containing 0.4 mL of 3.8% (wt/v) trisodium citrate as anticoagulant. For each donor, blood was centrifuged at 580 g for 8 min (BTI System V, BTI Biotechnology Institute, Spain) at room temperature. After centrifugation, the whole plasma column (PC) at the top of the tubes was obtained. The leukocyte buffy coat and the erythrocyte layer at the middle and bottom of the tubes respectively was avoided. PC was divided into three aliquots (PC1, PC2 and PC3) and maintained at room temperature until use. While the set up of the different in vitro assays was being prepared, the PC1 was activated with CaCl_2_ 10% [m/v] (PRGF-Activator, BTI Biotechnology Institute, Spain) and left to clot and retract at room temperature for a minimum of 40 min (20 µL CaCl_2_ 10% [m/v]: 1 mL PC1 ratio). This generated the supernatant of the first aliquot (SP1). At the moment of conducting each assay, PC2 was activated with a mixture of CaCl_2_ 10% [m/v] (20 µL CaCl_2_ 10% [m/v]:1 mL PC2 ratio) and SP1 (0.3 mL SP1:1 mL PC2 ratio) and left to clot and retract at room temperature for 2–3 min. This rapidly generated the supernatant of the second aliquot (SP2). Subsequently, 1 mL of PC3 and 0.3 mL of SP2 were loaded into two different syringes and injected simultaneously using a double syringe applicator (Nordson Medical, USA). This generated the E-sealant, a liquid formulation that rapidly gelated into an adhesive fibrin clot.

### Control sealant preparation

Tisseel® Frozen 4 mL (Baxter Healthcare Corp, Deerfield, USA) was used as the control sealant. Tisseel® is a ready to use formulation that is one of the most extended fibrin sealants in the market and stands as the gold standard for different applications (subsequently referred to as “Control”) [25]. Two different batches of Control were used for each assay during the experimental phase. At the time of conducting each assay, freshly prepared Control was obtained following the manufacturer´s instructions. Briefly, the fibrinogen component and the thrombin component that were preloaded in paralleling syringes were injected simultaneously using a double syringe applicator. This generated a viscous formulation that instantly gelated.

### Biological characterization

Before E-sealant preparation, several hematological parameters were measured in blood and in the whole plasma column using a hematology analyzer (Pentra ES-60, Horiba ABX, Montpellier, France). As Control is a non-autologous and commercially available product its biochemical composition can be checked in the manufacturer´s instructions.

### Coagulation kinetics

Coagulation kinetics was determined following changes in turbidity at 450 nm using a multimode microplate reader (Synergy H1, Agilent-Biotek, Santa Clara, USA). After injection at room temperature, the polymerization process was measured turbidimetrically every ten seconds. Assays were performed using plasma samples from three donors and technical triplicates.

### Microstructure analysis

For microstructure analysis, both formulations were histologically stained for haematoxylin-eosin (H&E) and may-grunwald-giemsa (MGG) (Sigma-Aldrich, St. Louis, USA). Briefly, clots were fixed in 10% formalin for 24 h, dehydrated and then embedded in paraffin. The resulting blocks were cut into 6 µm slices and stained for observation under optical microscope (Leica DMLB, Leica Microsystems, Wetzlar, Germany). Additionally, scanning electron microscopy (SEM) was employed to evaluate the ultrastructure of formulations. Samples were fixed with 2.5% glutaraldehyde, fixed with osmium tetroxide (1% OsO_4_ in 0.1 M cacodylate) and finally dehydrated through ascending alcohol concentrations. Thereafter, clots were subjected to critical point drying (Autosamdri 814, Tousimis, Rockville, USA), gold sputter coated and imaged using an electron microscope (S-4800, Hitachi, Japan).

### Rheological profile

A rheometer (Kinexus ultra+, Malvern Panalytical, Malvern, UK) was used to measure the rheological profile of both formulations. Rheological properties were recorded as a function of oscillatory frequency between 0.1 and 10 Hz and 0.5% shear strain at room temperature. The viscoelastic behavior of samples was determined over a shear stress range from 0.1 to 10 s^−1^. Assays were performed using plasma samples from three donors and technical triplicates.

### Biomechanical testing

Both formulations were subjected to tensile tests following the international standard of the American Society for Testing and Materials (ASTM) for tissue adhesives [26]. The sealants were applied over split thickness porcine skin grafts of 2.5 cm^2^, that were held by grips onto a mechanical testing station attached to a loading cell of 10 N (858 Mini Bionix II, MTS, USA). After sealant application onto the lower skin graft, confronted substrates were rapidly approached at a minimum gap and maintained for 2 min during sealant coagulation. Afterwards, a steady load at the vertical long axis was applied at a constant speed of 2 mm/min and the maximum load at failure was recorded. Assays were performed using plasma samples from five donors and five to ten technical replicates.

### Swelling/shrinkage assay

After clot formation, sealants were weighted and then incubated in phosphate buffered saline (PBS) at 37 °C to allow any swelling or shrinkage to occur. At different time points (1, 2, 4, 24, and 48 h) samples were removed from PBS and wet weight was measured. The swelling/shrinkage percentage at each time point was calculated compared to the initial weight. Assays were performed using plasma samples from three donors and technical triplicates were carried out.

### Biodegradation rate

Degradation upon exposure to tissue plasminogen activator (tPA) was studied over a period of 7 days by mass loss. In in vivo microenvironment, tPA converts plasminogen into plasmin which finally induces the degradation of fibrin. Briefly, sealant clots were immersed into whole plasma column (PC) of PRGF for 24 h at 37 °C. These were then weighted to obtain the initial weight. Afterwards they were transferred into new PC containing tPA (0.25 µg/ml, Abcam, Cambridge, UK) at 37 °C. Weight loss was recorded at timed intervals (1, 2, 3, 6 and 7 days). Assays were performed using plasma samples from three donors and technical triplicates were carried out.

### Statistical analysis

Statistical analysis is based on two-tail unpaired t-test. Results are reported as mean ± standard deviation. The statistical significance level was set as *p* < 0.05. Data were analyzed using GraphPad InStat 3.10 software.

## Results

### Biological characterization

Blood was obtained from three healthy male donors whose age ranged from 33 to 47 years-old (mean age 42 ± 8). Before E-sealant preparation, hematological content, fibrinogen content and prothrombin activity were determined within the whole plasma column. E-sealant presented a platelet-enriched content (431 ± 133 ×10^3^ platelets/µl) and practically no presence of leukocytes (0.17 ± 0.4 ×10^3^ leukocytes/µl). When compared to peripheral blood, the platelet enrichment reached 2.0 ± 0.4-fold. In contrast, Control contains no platelets nor leukocytes. Results are summarized in Table 1. According to manufacturer´s specifications, the fibrinogen component of Control included human fibrinogen (91 mg/mL), synthetic aprotinin (3000 UIC/mL), human albumin, L-histidine, niacinamide, polysorbate 80 and sodium citrate dihydrate. The thrombin component of Control included human thrombin (500 UI/mL), calcium chloride dihydrate (40 µmol/mL), human albumin, and sodium chloride.

**Table 1:** Hematological and biological content of E-sealant

Hematological content	
Platelets (×10^3^/µl)	431 ± 133
Platelet enrichment	2.0 ± 0.4
Leukocytes (×10^3^/µl)	0.17 ± 0.1
Fibrinogen (mg/dL)	381 ± 59
Prothrombin activity (%)	116 ± 11

### Coagulation kinetics

**Fig. 1:**
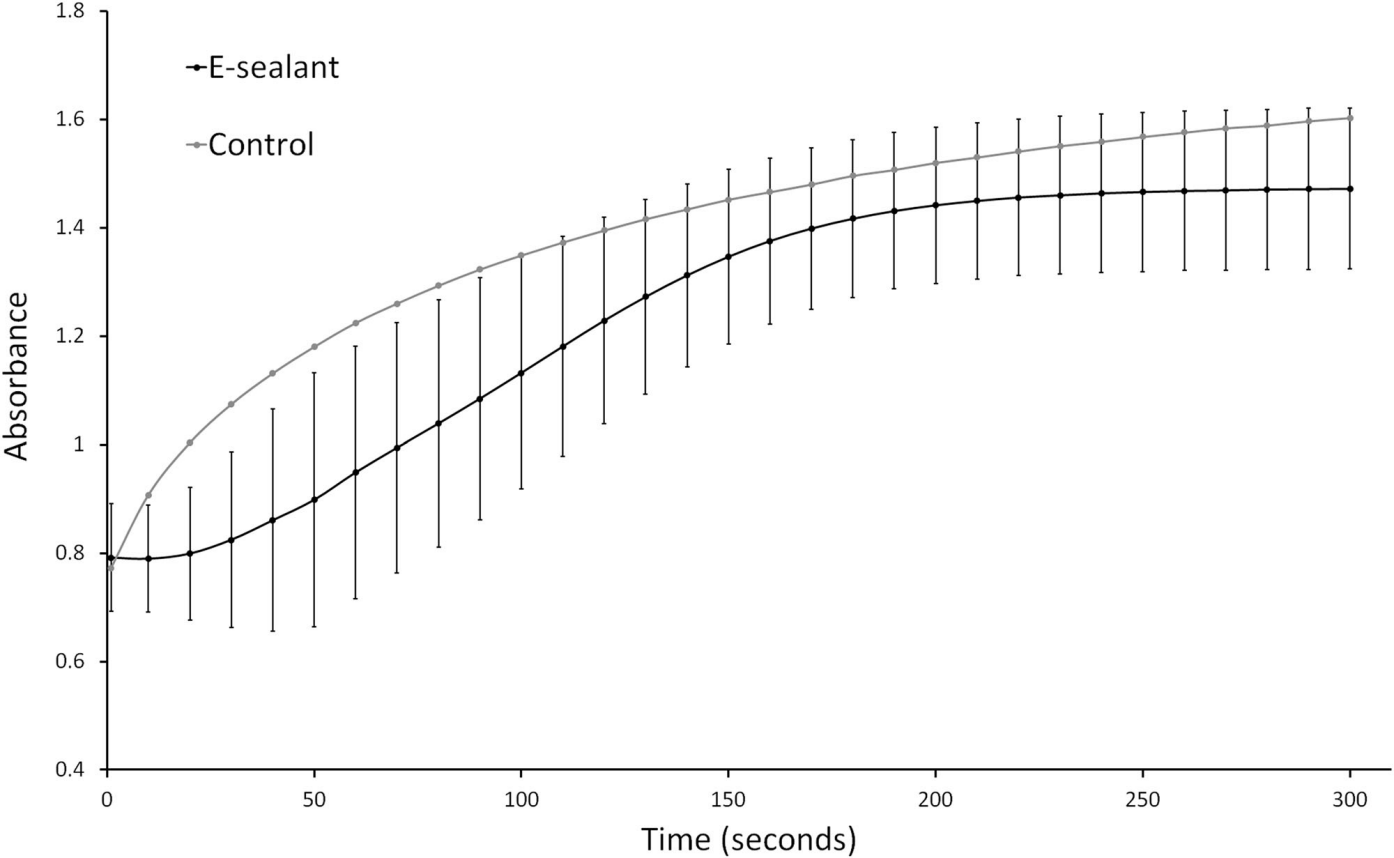
Coagulation assay results of both formulations after activation

The coagulation assay was performed using an automated microplate reader. The turbidity curve of Control showed immediate fiber formation with a virtually non-existent lag phase. In contrast, the protofibril formation within E-sealant lasted 30 s approximately before entering the subsequent lateral fiber aggregation phase. Despite from these differences in the coagulation dynamics, both formulations showed full coagulation three minutes after activation approximately (Fig. 1). The higher fibrinogen content of Control resulted in a denser fibrin clot compared to E-sealant, which was translated into higher absorbance values at each time point.

### Microstructure analysis

**Fig. 2:**
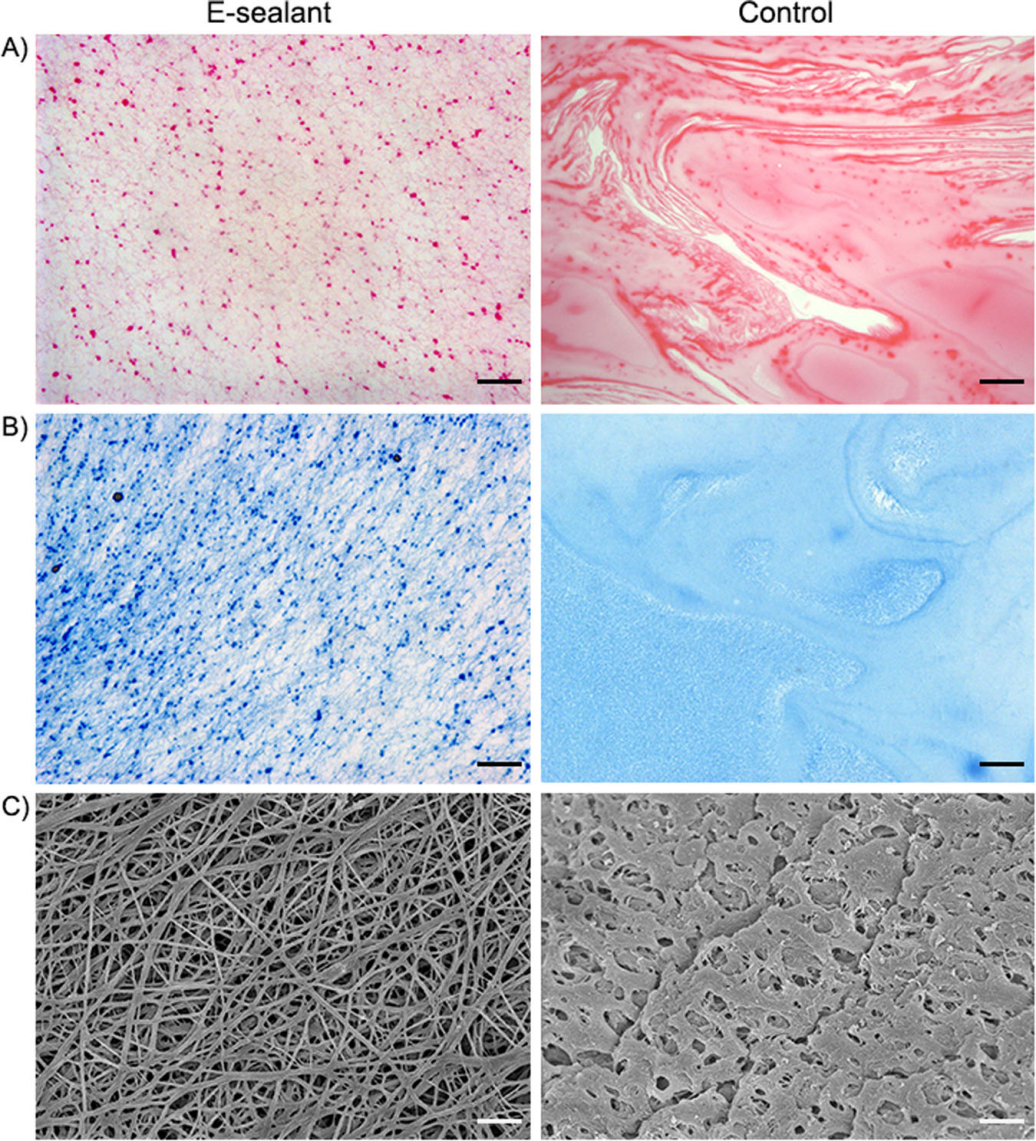
Histological examination of both formulations using H&E (**A**) and MGG (**B**) stained cross sections. High magnification view of the microstructure of the sealants using SEM imaging (**C**). Scale bars: 50 µm for H-E and MGG, and 10 µm for SEM

From a macroscopic point of view, E-sealant presented a yellowish semi-translucent gel appearance while Control polymerized uniformly into a coarse and opaque-pale clot. To observe the inner structure, stained cross sections were analyzed under a light microscope. H&E-stained E-sealant showed a fibrillar nature of the fibrin mesh and the formation of a homogeneous network along the sample. In contrast, Control presented a dense morphology with few interfibrillar pores and consistent protein deposits (Fig. 2A). MGG stain revealed that E-sealant presented numerous platelet aggregates scattered throughout intact fibrin strands, while the complete absence of blood cells was evident within Control samples (Fig. 2B). High magnification SEM images were used to analyze the microstructure in detail (Fig. 2C). E-sealant showed a highly porous fibrin scaffold with the absence of erythrocytes or leukocytes that might disrupt the autologous matrix. This network fitted and entangled in a three-dimensional mesh that provided irregular shaped spaces and pores with interconnected threads suitable for cell ingrowth. Control images revealed an amorphous hydrogel structure with coarse and poorly defined fibrillar networks that are clearly distinct from a physiological plasma clot and could hinder cell migration and growth.

### Rheological profile

**Fig. 3:**
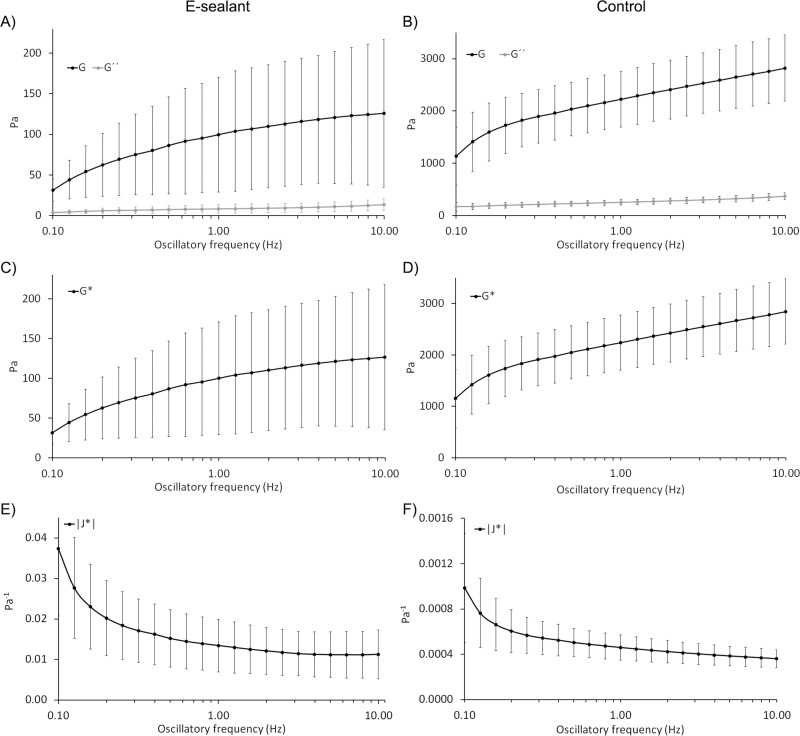
Rheological characterization of formulations. **A**, **B** Amplitude sweep data showing the storage modulus (G´) and the loss modulus (G´´). **C**, **D** A plot of the magnitude of the complex modulus (G*) and **E**, **F** the complex compliance |J*|

With the aim of determining the rheological profile, the formulations were subjected to shear stress after being extruded through application needles. Key parameters such as “storage modulus” (G´), “loss modulus” (G´´), and “complex modulus” (G*) were determined at 0.5% shear strain within an oscillatory frequency of 0.1–10 Hz. G´and G´´ of E-sealant ranged from 31 ± 13 Pa to 126 ± 91 Pa and from 3.4 ± 1.3 Pa to 13.4 ± 6.9 Pa respectively (Fig. 3A). G´and G´´ of Control ranged from 1139 ± 557 Pa to 2821 ± 631 Pa and from 168 ± 88 Pa to 371 ± 67 Pa respectively (Fig. 3B). As E-sealant is an autologous formulation, G´and G´´ values remained in a lower order of magnitude compared to Control, which has a supraphysiological level of fibrinogen. However, these results evidenced that both formulations shared a viscoelastic nature and had the ability to partially recover their original shape after deformation. The stiffness of the formulations (G*), reached values from 32 ± 14 Pa to 127 ± 91 Pa for E-sealant and 1152 ± 564 Pa to 2845 ± 634 Pa for Control (Fig. 3C, D). Similarly, the “complex compliance” (|J*|) that measures the deformability of a material, ranged from 0.04 ± 0.01 Pa^−1^ to 0.01 ± 0.006 Pa^−1^ and 9.8 ×10^−4^ ± 4.8 ×10^−4 ^Pa^−1^ to 3.7 ×10^−4^ ± 8 ×10^−5 ^Pa^−1^ for E-sealant and Control respectively (Fig. 3E, F). These results evidenced that Control formulation presented a higher hardness and lower deformability compared to E-sealant.

**Fig. 4:**
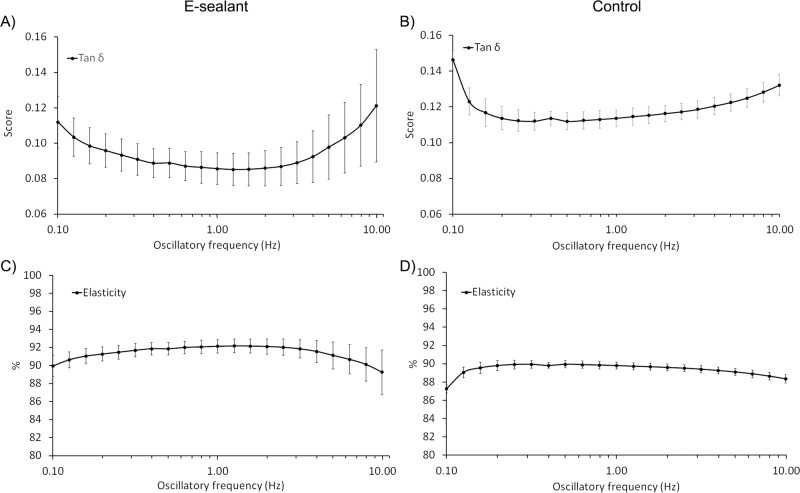
Rheological characterization of formulations. **A**, **B** Amplitude sweep data showing the tan δ score and **C**, **D** the percent elasticity within formulations

**Fig. 5:**
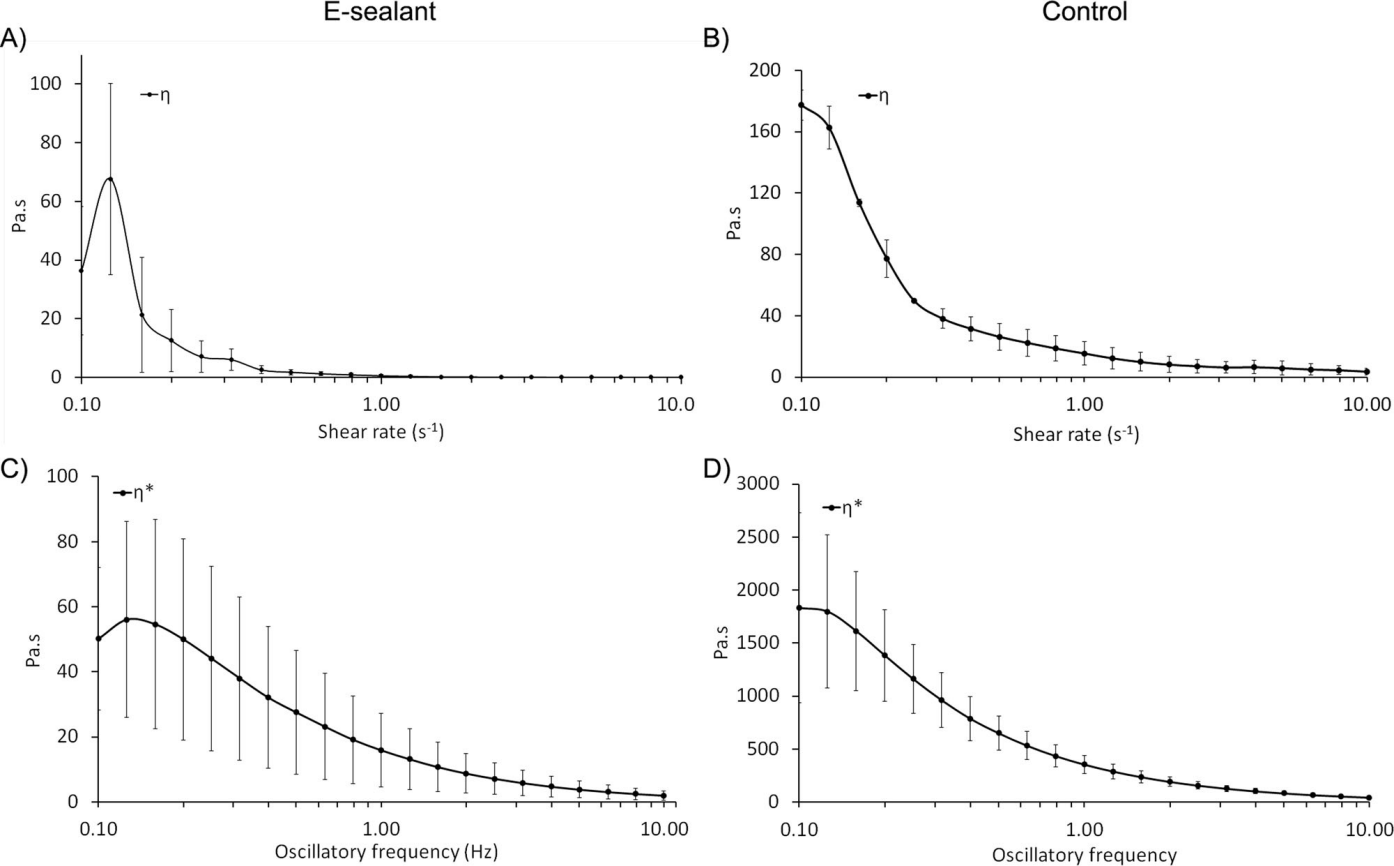
Rheological characterization of formulations. **A**, **B** A plot of the magnitude of the dynamic viscosity (η). **C**, **D** Amplitude sweep data showing the complex viscosity (η*)

The viscoelasticity (Tan δ) and the “percent elasticity” (%) of the sealants were also evaluated. Both formulations presented an elastic behavior rather than a viscous gel behavior, as Tan δ values remained in the range of 0 to 1 score (Fig. 4A, B). Moreover, E-sealant showed a slightly higher proportion of elasticity behavior within the formulation compared to Control (Fig. 4C, D). Both dynamic viscosity (η) and complex viscosity (η*) of formulations were additionally determined (Fig. 5). While dynamic viscosity is a measure of a fluid’s resistance to flow over a shear rate (0.10 to 10 s^−1^), complex viscosity measures the ability to store and dissipate energy under shear strain deformation within an oscillatory frequency. E-sealant´s η and η* values remained in a lower order of magnitude because Control has a supraphysiological level of viscous fibrinogen. However, these results evidenced that both formulations resembled a pseudoplastic hydrogel that remained in the site of application after extrusion rather that spreading out. Additionally, they also showed a typical shear thinning behavior that is clinically appropriate for topical use over tissue surface. Table 2 summarizes and compares the rheological properties of both formulations.

**Table 2:** Rheological properties of formulations at an oscillatory frequency of 1 Hz, 0.5% strain and 1^−1^ of shear rate

Rheology	E-sealant	Control
G´ (Pa)	100 ± 71	2226 ± 535
G´´ (Pa)	8 ± 5	252 ± 50
G* (Pa)	100 ± 71	2240 ± 537
J* (Pa^−1^)	0.013 ± 0.006	0.0005 ± 0.0001
Tan δ	0.086 ± 0.009	0.114 ± 0.005
elasticity (%)	92 ± 0.7	90 ± 0.4
η (Pa.s)	0.56 ± 0.43	16 ± 8
η* (Pa.s)	16 ± 11	357 ± 85

### Biomechanical testing

**Fig. 6:**
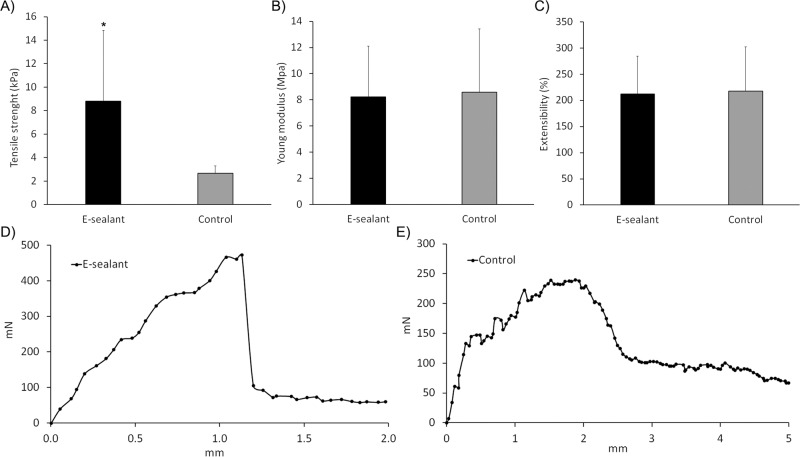
Biomechanical tensile test of formulations. **A** Tensile strength. **B** Young´s modulus. **C** Extensibility. **D**, **E** Tensile curve profiles. * Statistically significant differences between sealants (*p* < 0.05)

To simulate the mechanical dehiscence force that a sealant might encounter in vivo, both formulations were subjected to tensile tests. Regarding the maximum load at failure, E-sealant presented a significantly higher tensile strength compared to Control (8.8 ± 6 kPa and 2.7 ± 1 kPa respectively) (*p* < 0.05) (Fig. 6A). Both formulations showed a cohesive failure profile which refers to a break within the sealant itself, leaving behind fibrin residues on both confronted skin substrates. This is related to an acceptable adhesion behavior within tissue sealants.

Additionally, the Young´s modulus and the extensibility of the formulations were calculated in the linear region before reaching the maximum load at the failure point. Both sealants presented similar results regarding their elastic stretching behavior as a Young´s modulus of 8.22 ± 3.9 MPa and 8.60 ± 4.8 MPa for E-sealant and Control were achieved respectively (Fig. 6B). Accordingly, the maximum elongation with respect to full adherence at baseline, was also similar for both formulations (212 ± 72% and 218 ± 85% for E-sealant and Control respectively) (Fig. 6C). However, the tensile curve profiles showed slight differences between both formulations. E-sealant presented a steady load increase until reaching the break point that was followed by a sudden and complete tensile force drop. This resembled a clean cohesive failure in which the sealant did no longer exert an adhesive force (Fig. 6D). In contrast, the tensile force after Control failure showed a progressive loss of adherence rather than a total drop of the adhesive strength (Fig. 6E). This could be related to isolated fibrin threads that were macroscopically observable and remained attached to the confronted substrates after failure, which still could exert residual tensile strength.

### Swelling/shrinkage assay

**Fig. 7:**
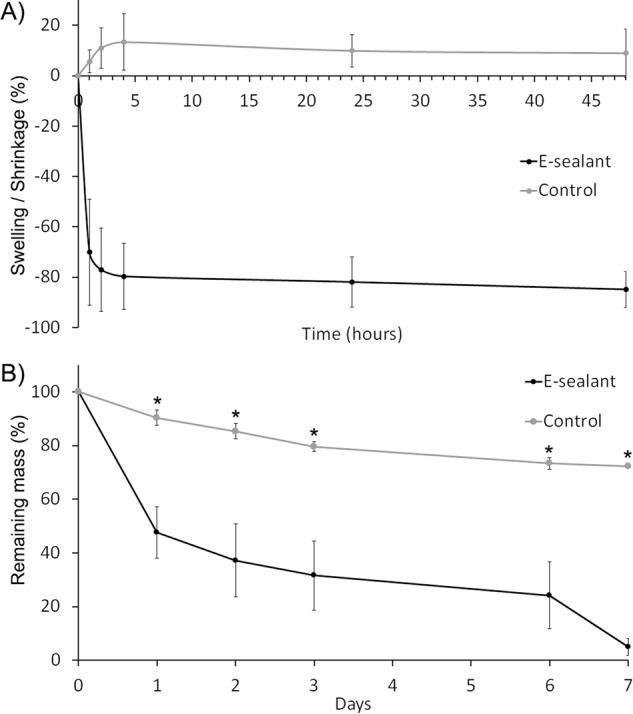
**A** Swelling/shrinkage percentage of formulations. **B** Biodegradation rate of formulations. * Statistically significant differences between sealants (*p* < 0.05)

The swelling/shrinkage assay was performed to simulate the in vivo behavior of sealants where tissue fluids are abundant and ubiquitous at the application site. E-sealant and Control showed different behaviors (Fig. 7A). While E-sealant presented a significant shrinkage process (84 ± 7% after 48 h), Control showed a slight swelling over time (8.9 ± 9% after 48 h). These phenomena took place mainly in the first four hours after the beginning of the assay and were stabilized for two days.

### Biodegradation rate

Both formulations were incubated with tPA, the enzyme that converts the plasminogen into plasmin, to simulate their resorption profile in a catalytic in vivo microenvironment (Fig. 7B). E-sealant presented a high resorption rate as only the 5 ± 3% of the mass remained after seven days of tPA incubation. In contrast, Control showed to withstand the biodegradation process in a significant way as 72 ± 1% of the mass was detected at one week (*p* < 0.05).

## Discussion

The use of tissue adhesives is gaining scientific and clinical interest to enhance wound closure that has been catalyzed by the increase of the number of surgeries performed every year (more than 300 million surgeries) [1]. Their use is aimed at avoiding complications and promote wound healing. For that, they need to be biocompatible and biodegradable, and have appropriate adhesive properties and sufficient mechanical stability to withstand tissue forces [27]. Depending on the adhesive and mechanical properties, tissue adhesives could be intended to be an alternative or an adjuvant to wound suturing.

Fibrin-based adhesive or sealants have an important advantage over other adhesive systems that are based on cyanoacrylates or marine materials. This advantage is its biocompatibility, that make them widely used in the clinical practise and in different tissues. However, most of them are based on pooled plasma of several donors and this may restrict their application [28]. For that, the development of an equivalent fibrin-based adhesive of autologous origin would ease the restrictions on its use. Moreover, the allogeneic fibrin-based systems lack the biological activity of proteins and growth factors found within PRP secretome.

In this study, both autologous and allogeneic adhesives had higher elastic behavior than viscous properties. In comparison to Tisseel^®^, E-sealant had an overall lower resistance to deformation which is indicative of better spreading at the tissue interface. This would influence the higher tissue bonding of E-sealant in comparison to Tisseel^®^ under tensile force, where the wound interface is separated away. The tensile strength of the autologous and allogeneic fibrin sealants was 8.8 ± 6 kPa and 2.7 ± 1 kPa respectively. Other studies have reported similar values for the Tisseel^®^ (approximately 5 kPa) [29]. These results suggest that E-sealant has a comparable adhesive strength to Tisseel^®^. Both suffered from a cohesive failure (within the adhesive) indicating their good adhesion to the skin samples. These data would suggest an adjuvant use of E-sealant to wound suturing or as topical spray. The adhesive strength of allogeneic fibrin sealants is dependent on fibrinogen concentration which is in an order of magnitude higher than autologous PRP (physiological concentration) [12]. In an interesting study, similar adhesive strength was observed between autologous fibrinogen that was mixed with allogeneic thrombin and commercially available fibrin sealants [6, 12]. Thus, the fibrinogen concentration in autologous preparations could be sufficient to develop 100% autologous fibrin adhesives [6, 12, 30]. Data from this study support this assumption however, it needs to be assessed in other tissue types.

In this study, the autologous E-sealant had higher degradation rate than the allogeneic sealant. One explanation of this is the presence of antifibrinolytic components in most of allogeneic fibrin sealants. For fast wound closure, this is an important factor that could affect the clinical suitability of the autologous fibrin sealant. In vivo and clinical evidence have shown the positive effect of PRGF to boost tissue healing and wound closure in different tissues like tooth extraction socket, mucocutaneous ulcers, bone grafting and cartilage grafting [31–36]. A direct comparison of PRGF to allogeneic fibrin sealants have shown similar hemostatic properties of both formulations but faster wound closure in PRGF [37]. The creation of a dense fibrin clot in the allogeneic sealants could hinder cell migration and extracellular matrix deposition [12]. Meanwhile, PRGF has a more porous scaffold that promotes cell migration, proliferation, and ECM formation [38, 39]. The fibrin clot also acts as a system that localizes PRGF-derived healing cues at the wound site and promote their progressive release, assuring their availability to boost healing [20, 40, 41]. Thus, the faster degradation of the E-sealant could be balanced by faster wound healing.

This study has several limitations that need to be considered. PRGF is a leukocyte-free platelet-rich plasma with moderate platelet concentration and thus the effect of the cellular composition on the performance of the fibrin glue has not been assessed. There is a need for future studies to assess the composition effect as several studies have reported that leukocytes would hinder the stability of the fibrin clot. Moreover, the presence of leukocytes and erythrocytes could alter the mechanical and rheological properties of the fibrin adhesive [38, 42]. Moreover, cell proliferation and deposition of the extracellular matrix has shown to be altered by the inclusion of blood cells into PRP [38, 43]. Another limitation is that fibrinogen and thrombin concentration within E-sealant have not been altered, thus limiting the adhesive potential of the formulation. However, this has a significant impact on the easy preparation of the adhesive. Moreover, there is a need to validate these results in an in vivo model.

In summary, the use of physiological concentration of fibrinogen and thrombin has resulted in an autologous fibrin sealant with similar adhesive strength to an allogeneic fibrin glue. Thus, E-sealant presents optimal biochemical and biomechanical properties suitable for its use in regenerative medicine.
